# Does preoperative bone marrow edema affect clinical outcomes after medial open-wedge high tibial osteotomy?

**DOI:** 10.1186/s43019-023-00200-7

**Published:** 2024-02-12

**Authors:** Hee-June Kim, Ji-Yeon Shin, Hyun-Joo Lee, Dong-Hyun Kim, Yeonggwon Jo, Joon-Woo Kim, Chang-Wug Oh, Hee-Soo Kyung

**Affiliations:** 1 Department of Orthopaedic Surgery, School of Medicine, Kyungpook National University, Kyungpook National University Hospital, 130 Dongduk-Ro Jung-Gu, Daegu, 41944 Korea; 2 Department of Preventive Medicine, School of Medicine, Kyungpook National University, Daegu, Korea

**Keywords:** Osteotomy, Knee, Magnetic resonance imaging

## Abstract

**Purpose:**

The aim of this study is to evaluate the results of high tibial osteotomy (HTO) in patients with bone marrow edema (BME) and assess the effect of factors on the subsequent results.

**Methods:**

A total of 138 patients who underwent medial open-wedge HTO using locking plate were included in this study. BME was observed in 108 patients using preoperative magnetic resonance imaging. Clinical results were evaluated before HTO and postoperatively at 12 months. Moreover, we evaluated the factors affecting the clinical results.

**Results:**

The clinical scores were all significantly improved regardless of the presence of BME. There were no differences in improvement of clinical scores between patients with BME and without BME. Patients with BME showed higher postoperative Western Ontario and McMaster Universities Arthritis Index (WOMAC) stiffness scores than patients without BME. Furthermore, patients with BME on both the femur and tibia showed lower Knee Society function scores than patients with BME on either the femur or the tibia. Patients with large BME lesions exhibited a lower Hospital for Special Surgery score and WOMAC pain scores, postoperatively. In patients with BME, patients with undercorrection showed significantly lower improvement in WOMAC pain scores compared with patients with acceptable correction.

**Conclusions:**

The clinical improvement after HTO in patients with varus and medial osteoarthritis was not different regardless of the presence or absence of BME. However, accurate alignment should be considered essential for achieving better clinical outcomes in patients with preoperative BME.

## Introduction

High tibial osteotomy (HTO) is an effective treatment option for medial osteoarthritis (OA) with varus deformity, especially in young (< 65 years old), active patients [1–5]. This surgical procedure can reduce abnormal loads in the medial compartment by correcting the mechanical axis to the lateral compartment [4, 6–8]. Furthermore, this operation can shift load bearing from the medial compartment to lateral compartments that are less affected, a method that has been shown to facilitate cartilage regeneration [9]. These good clinical results have been reported by several studies following HTO [10, 11].

Bone marrow edema (BME) is a nonspecific finding characterized by an ill-defined marrow area of T1-weighted images with low signal intensity and hyperintensity on short tau inversion recovery images and on T2-weighted, fat-suppressed magnetic resonance imaging (MRI) [12, 13]. This condition can be diagnosed in patients with trauma, stress fracture, osteonecrosis, and reactive OA [12, 14]. Especially, BME of the knee joint can be observed in patients even with mild OA and with varus deformities due to the effect of overloading on the subchondral bone.

In patients with varus deformities and medial knee OA, HTO can be an effective treatment modality. Therefore, good clinical results can be obtained in patients with BME, and medial OA could also be treated using HTO. Moreover, the extent of BME or postoperative alignment can affect the postoperative clinical results. The aim of this study is to evaluate how BME could affect the clinical results of patients who underwent HTO. Moreover, this study evaluated the correlation between the postoperative results and the factors including the location, extent, or volume of preoperative BME and postoperative alignment.

## Patients and methods

In this study, 138 patients (31 men and 107 women) who underwent medial open-wedge HTO owing to varus deformity and medial OA were selected between October 2010 and December 2019 with a mean age of 58.6 (35–70) years. Among our subjects, BME was observed in 108 patients using preoperative MRI. All patients underwent open-wedge HTO with locking plates. TomoFix locking plates (Synthes GmbH, Solothurn, Switzerland) were used in 62 patients and OhtoFix locking plates (Ohtomedical Co., Ltd., Goyang, Korea) were used in 76 patients. The inclusion criteria were as follows: (1) age ≤ 70 years, (2) body mass index (BMI) ≤ 30 kg/m^2^, (3) only medial-side pain, (4) sports activity except running and jumping, (5) varus angle < 15°, and (6) < 10° of knee extension loss and > 90° of knee flexion. The exclusion criteria were as follows: (1) flexion contracture > 25°, (2) bicompartmental disease, (3) elderly patients with difficulty mobilizing, and (4) BMI > 30 kg/m^2^. There were no differences in demographics, including age, sex, and preoperative varus deformity between patients with or without BME (Table 1).

**Table 1:** Preoperative demographics and clinical scores in both groups

	BME	No BME	*p*-Value
Age (years)	58.4 ± 4.4 (35–68)	59.0 ± 4.7 (49–70)	0.543
Sex (male:female)	25:83	6:24	0.715
Mechanical axis (%)	14.8 ± 12.6 (−15.5 to 46.1)	11.8 ± 11.6 (−20.5 to 37.4)	0.236
HSS score	73.4 ± 10.4 (41–87)	73.5 ± 8.2 (44–85)	0.960
Knee Society knee score	73.3 ± 11.1 (47–86)	76.6 ± 8.4 (61–89)	0.130
Knee Society function score	59.7 ± 15.0 (10–80)	61.3 ± 10.7 (40–90)	0.572
WOMAC score	42.1 ± 16.4 (10–85)	39.8 ± 11.9 (14–57)	0.406
Pain	9.9 ± 3.6 (2–18)	9.6 ± 2.6 (3–15)	0.567
Stiffness	3.0 ± 1.7 (0–7)	3.1 ± 1.7 (1–7)	0.825
Function	29.2 ± 12.5 (7–62)	27.2 ± 9.4 (9–47)	0.420

*BME* bone marrow edema, *HSS* Hospital for Special Surgery, *WOMAC* Western Ontario and McMaster Universities Arthritis Index

This study was approved by the institutional review board (IRB) of our institution (IRB no. 2021-06-023).

### Surgical technique

The patients underwent full-length anteroposterior lower limb weight-bearing radiography preoperatively. The target point was determined to be the mechanical axis of the limb located at the 62.5% point from the medial border along the longest medial-to-lateral width of the tibial plateau.

A single surgeon performed all surgeries. A proximal biplanar osteotomy was performed behind the tibial tuberosity after the pes anserinus was completely separated, and the superficial medial collateral ligament was elevated from the periosteum. Thereafter, the posteromedial tibial osteotomy site was opened, and locking plate fixation was performed. Allogeneic bone chips (ReadiGRAFT Cancellous Chips, LifeNet Health, Virginia Beach, Virginia) mixed with autologous bone marrow were harvested from the ipsilateral anterior superior iliac spine and were grafted into the osteotomy gap. The osteotomy site was covered with the superficial medial collateral ligament, and the pes anserinus was resutured to the periosteal membrane.

All patients performed range of motion activities from the second day with maintenance of a night splint. A partial weight bearing was permitted from 2 weeks postoperatively, whereas full weight bearing with a brace was allowed after 4 weeks.

### Assessment

Clinical results, including the Hospital for Special Surgery (HSS) score, Knee Society knee score (KS), and function score (FS), and The Western Ontario and McMaster Universities Arthritis Index (WOMAC), were evaluated before HTO and postoperatively at 12 months. Moreover, we evaluated the factors affecting the clinical results, including the existence, position, extent, and volume of BME and postoperative correction degree.

**Fig. 1:**
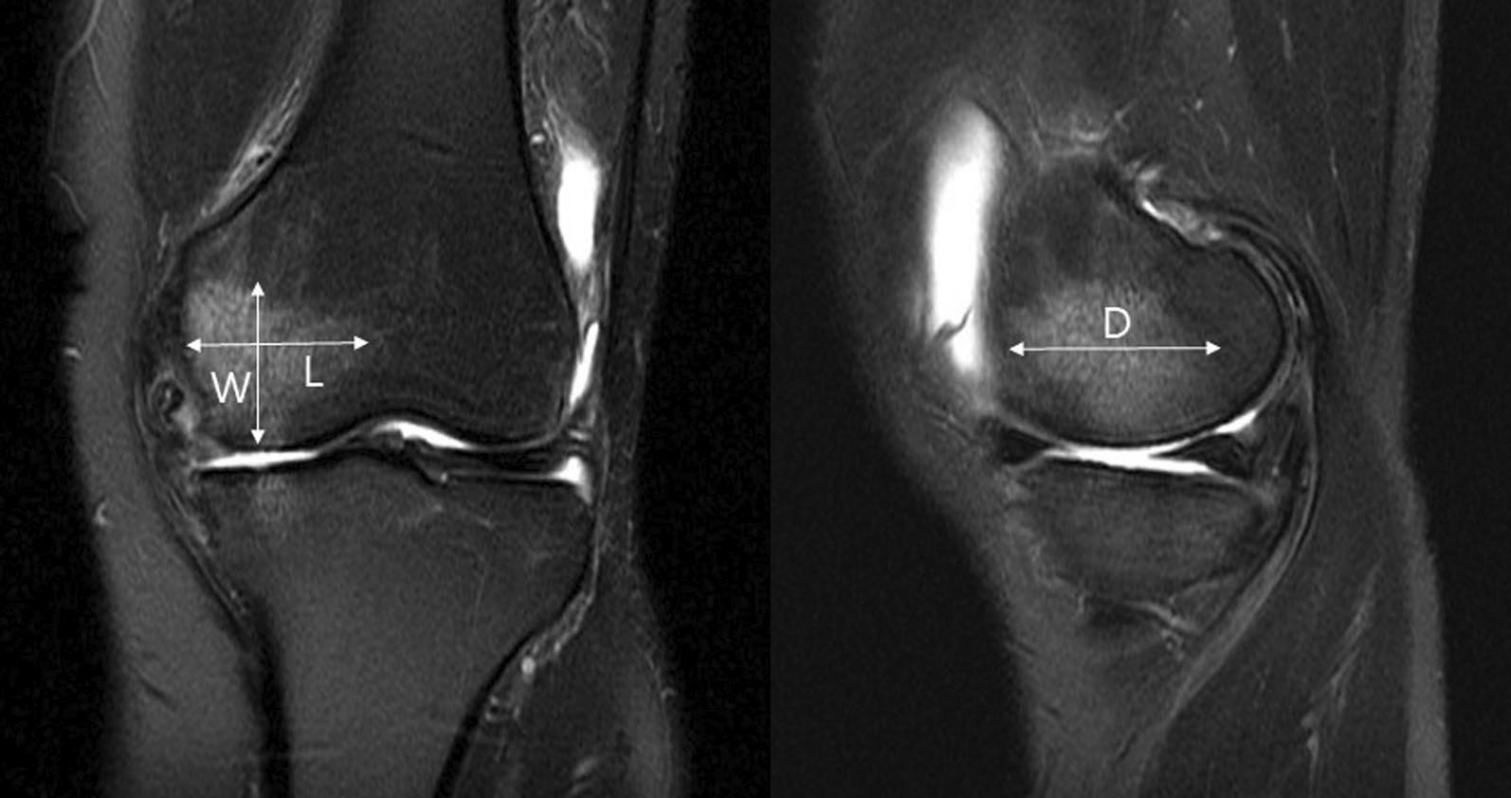
A preoperative coronal and sagittal MR image of a 52-year-old woman shows subchondral BME in the medial femoral condyle. The greatest lateral (L = length) and cranial (W = width) extents were measured on coronal images and the greatest sagittal extent (D = depth) was measured on sagittal images. The volumes were calculated using the formula for a prolate ellipsoid: length × width × depth × *π*/6

Patients underwent preoperative MRI to evaluate BME, and all planes (coronal, sagittal, and axial) and T1 and T2-weighted sequences were reviewed in a picture archiving and communication system. BME on MRI was characterized by an ill-defined marrow area of T1-weighted images with low signal intensity and hyperintensity on short tau inversion recovery images and on T2-weighted, fat-suppressed MRI. Furthermore, the existence, position, extent, and volume of BME were evaluated using MRI. BME was all in the weight-bearing area. The position of BME was divided into groups of (a) only femur, (b) only tibia, and (c) both femur and tibia. The extent of BME was evaluated in accordance with the report of Welsch et al. [15]. The maximum BME diameter scores were determined as follows: 1 = small (< 1 cm), 2 = medium (< 2 cm), 3 = large (< 4 cm), and 4 = very large (> 4 cm). The scores of each tibia and femur were summarized and divided into two groups. The large group was defined as the summarized scores being 5 or more, and the small group being 4 or less. The volume of BME was calculated using the formula for prolate ellipsoid: length × width × depth × *π*/6(Fig. 1) [16]. The median value of the volume was 2.98 cm^3^, and groups were classified according to this value.

The alignment of the patients was evaluated using full-length lower limb radiographs. The percentages of crossing points of the weight-bearing line and tibial plateau with respect to the medial border were measured. As mentioned above, the target point was defined the 62.5% point from the medial border along the longest medial-to-lateral width of the tibial plateau. The postoperative correction degree was divided into three types. Acceptable correction was determined to be within 5% of the target point (57.5–67.5%), undercorrection was determined to be under 5% of the target point (57.5%), and overcorrection was determined to be over 5% of the target point (67.5%) [17].

### Statistical analysis

The paired *t*-test was used to compare preoperative and postoperative values. Analysis of variance (ANOVA) test and independent *t*-test were used for evaluation of the factors, including the existence, position, extent, and volume of BME. ANOVA test was used for evaluating the differences of clinical results based on postoperative correction acceptability. Moreover, Scheffé’s post hoc analysis was performed to confirm the difference among groups.

Statistical significance was assumed at a *p* value < 0.05. All statistical analyses were performed using SPSS Statistics version 21.0 (IBM Corp., Armonk, NY).

## Results

Alignment was corrected from 14.1 ± 12.4% (−20.5% to 46.1%) preoperatively to 58.3 ± 6.7% (45.9–73.9%) postoperatively after medial open-wedge HTO in a total of 138 patients. All clinical scores were significantly improved in both groups, 103 patients with BME and 30 patients without BME (Table 2). The mean improvement of all scores was not different between two groups (Table 3). There were not postoperative complications including the fracture, nonunion, or infection.

**Table 2:** Preoperative and postoperative results in both groups

		Preoperative	Postoperative	*p*-Value
Mechanical axis (%)	BME	14.1 ± 12.6 (−15.5 to 46.1)	58.7 ± 6.8 (41.3–73.9)	< 0.001
	No BME	11.8 ± 11.6 (−20.5 to 37.4)	56.8 ± 6.2 (45.9–71.6)	< 0.001
HSS score	BME	73.4 ± 10.4 (41–87)	91.4 ± 4.3 (76–98)	< 0.001
	No BME	73.5 ± 8.2 (44–85)	92.9 ± 3.4 (86–99)	< 0.001
Knee Society knee score	BME	73.3 ± 11.1 (47–86)	94.5 ± 4.3 (70–100)	< 0.001
	No BME	76.6 ± 8.4 (61–89)	96.1 ± 2.9 (90–100)	< 0.001
Knee Society function score	BME	59.7 ± 15.0 (10–80)	88.1 ± 7.6 (70–100)	< 0.001
	No BME	61.3 ± 10.7 (40–90)	90.7 ± 5.8 (80–100)	< 0.001
WOMAC score	BME	42.1 ± 16.4 (10–85)	10.9 ± 4.4 (2–20)	< 0.001
	No BME	39.8 ± 11.9 (14–57)	9.1 ± 3.6 (2–16)	< 0.001
Pain	BME	9.9 ± 3.6 (2–18)	3.4 ± 1.7 (0–9)	< 0.001
	No BME	9.6 ± 2.6 (3–15)	2.8 ± 1.2 (1–5)	< 0.001
Stiffness	BME	3.0 ± 1.7 (0–7)	0.9 ± 0.8 (0–3)	< 0.001
	No BME	3.1 ± 1.7 (1–7)	0.6 ± 0.7 (0–2)	< 0.001
Function	BME	29.2 ± 12.5 (7–62)	6.6 ± 3.0 (1–14)	< 0.001
	No BME	27.2 ± 9.4 (9–47)	5.8 ± 2.7 (0–11)	< 0.001

*BME* bone marrow edema, *HSS* Hospital for Special Surgery, *WOMAC* Western Ontario and McMaster Universities Arthritis Index

**Table 3:** Postoperative clinical improvement (change in score) according to the presence or absence of bone marrow edema

	BME	No BME	*p*-Value
HSS score	18.1 ± 10.0 (0–57)	19.4 ± 8.9 (−10 to 50)	0.502
Knee Society knee score	21.2 ± 10.7 (0–45)	19.5 ± 9.5 (−1 to 39)	0.436
Knee Society function score	28.4 ± 14.7 (3–55)	29.3 ± 12.6 (−10 to 50)	0.751
WOMAC score	31.2 ± 15.3 (4–79)	30.7 ± 12.0 (4–51)	0.861
Pain	6.6 ± 3.4 (−1 to 16)	6.8 ± 2.4 (1–11)	0.648
Stiffness	2.1 ± 1.5 (−1 to 5)	2.5 ± 1.7 (0–6)	0.196
Function	22.6 ± 12.7 (−1 to 56)	21.4 ± 9.6 (1–39)	0.619

*BME* bone marrow edema, *HSS* Hospital for Special Surgery, *WOMAC* Western Ontario and McMaster Universities Arthritis Index

Patients with BME on both femur and tibia showed lower FS than patients with BME on either femur or tibia (*p* = 0.045) (Table 4). Pertaining to the BME extent, patients with large BME lesions exhibited lower HSS and WOMAC pain scores, postoperatively (*p* = 0.030 and *p* = 0.039, respectively) (Table 5). With respect to the BME volume, there were no significant differences in clinical scores between patients with high and low volume (Table 6).

**Table 4:** Postoperative clinical results according to the bone marrow edema location

	BME on either femur or tibia	BME on both femur and tibia	*p*-Value
HSS score	91.7 ± 3.9 (85–99)	91.4 ± 4.4 (76–99)	0.768
Knee Society knee score	95.1 ± 4.3 (81–100)	94.3 ± 4.3 (70–100)	0.400
Knee Society function score	90.9 ± 6.0 (80–100)	87.3 ± 7.8 (70–100)	0.045
WOMAC score	10.5 ± 4.6 (3–18)	11.0 ± 4.4 (2–20)	0.649
Pain	3.4 ± 1.7 (1–8)	3.4 ± 1.7 (0–9)	0.967
Stiffness	1.0 ± 0.7 (0–2)	0.9 ± 0.8 (0–3)	0.472
Function	6.1 ± 3.1 (2–12)	6.7 ± 2.9 (1–14)	0.402

*BME* bone marrow edema, *HSS* Hospital for Special Surgery, *WOMAC* Western Ontario and McMaster Universities Arthritis Index

**Table 5:** Postoperative clinical results according to the bone marrow edema extent

	Small size	Large size	*p*-Value
HSS score	92.2 ± 3.8 (85–98)	90.4 ± 4.7 (76–98)	0.030
Knee Society knee score	95.1 ± 3.6 (81–100)	93.7 ± 5.0 (70–100)	0.096
Knee Society function score	89.2 ± 6.2 (70–100)	86.7 ± 8.8 (70–100)	0.104
WOMAC score	10.3 ± 4.0 (3–19)	11.5 ± 4.8 (2–20)	0.153
Pain	3.1 ± 1.6 (0–8)	3.7 ± 1.8 (0–9)	0.039
Stiffness	0.9 ± 0.7 (0–2)	0.9 ± 0.8 (0–3)	0.998
Function	6.3 ± 2.8 (2–13)	6.9 ± 3.2 (1–14)	0.332

*HSS* Hospital for Special Surgery, *WOMAC* Western Ontario and McMaster Universities Arthritis Index

**Table 6:** Postoperative clinical results according to the bone marrow edema volume

	Small volume	Large volume	*p*-Value
HSS score	92.1 ± 3.9 (85–98)	90.7 ± 4.6 (75–98)	0.085
Knee Society knee score	94.7 ± 3.5 (81–100)	94.2 ± 5.0 (70–100)	0.521
Knee Society function score	89.5 ± 6.0 (80–100)	86.7 ± 8.7 (70–100)	0.053
WOMAC score	10.6 ± 3.7 (3–18)	11.2 ± 5.0 (2–20)	0.487
Pain	3.2 ± 1.6 (0–8)	3.5 ± 1.8 (0–9)	0.468
Stiffness	0.9 ± 0.8 (0–2)	0.9 ± 0.8 (0–3)	0.900
Function	6.4 ± 2.6 (2–13)	6.8 ± 3.3 (1–14)	0.562

*HSS* Hospital for Special Surgery, *WOMAC* Western Ontario and McMaster Universities Arthritis Index

According to postoperative alignment, the postoperative clinical scores were not different in both patients with and without BME (Table 7). However, in patients with BME, the change of WOMAC pain score was correlated to the degree of correction (*p* = 0.016). Especially, in patients with BME, patients with undercorrection showed significantly lower improvement compared with patients with acceptable corrections (*p* = 0.019) (Table 8).

**Table 7:** Postoperative clinical results according to alignment in patients with BME

Postoperative score	Undercorrection (*n* = 42)	Acceptable correction (*n* = 59)	Overcorrection (*n* = 7)	*p*-Value
HSS score	91.7 ± 3.9 (83–98)	91.1 ± 4.6 (75–98)	92.2 ± 4.1 (86–98)	0.682
Knee Society knee score	95.0 ± 3.9 (81–100)	94.1 ± 4.7 (70–100)	94.1 ± 3.4 (91–100)	0.581
Knee Society function score	89.3 ± 7.1 (70–100)	87.3 ± 8.1 (70–100)	87.1 ± 4.9 (80–90)	0.388
WOMAC score	10.6 ± 4.1 (4–61)	10.9 ± 4.7 (8–79)	11.7 ± 4.2 (16–52)	0.834
Pain	3.5 ± 1.7 (1–9)	3.3 ± 1.8 (0–8)	3.0 ± 1.0 (2–5)	0.727
Stiffness	0.8 ± 0.7 (0–2)	0.9 ± 0.8 (0–3)	1.1 ± 1.1 (0–3)	0.497
Function	6.3 ± 2.6 (2–14)	6.7 ± 3.2 (1–13)	7.6 ± 3.0 (3–12)	0.577

*HSS* Hospital for Special Surgery, *WOMAC* Western Ontario and McMaster Universities Arthritis Index

**Table 8:** Postoperative clinical results (change in score) according to alignment in patients with BME

Change in score	Undercorrection (*n* = 42)	Acceptable correction (*n* = 59)	Overcorrection (*n* = 7)	*p*-Value
HSS score	16.8 ± 9.7 (0–40)	19.2 ± 10.5 (0–57)	16.3 ± 6.4 (8–27)	0.444
Knee Society knee score	18.9 ± 10.0 (0–40)	23.1 ± 10.6 (4–45)	18.4 ± 13.0 (4–40)	0.121
Knee Society function score	26.1 ± 15.1 (0–55)	29.3 ± 15.0 (0–50)	34.3 ± 5.3 (3–40)	0.309
WOMAC score	28.0 ± 15.0 (4–61)	33.5 ± 15.7 (8–79)	32.1 ± 10.7 (16–52)	0.202
Pain	5.4 ± 3.0 (−1 to 12)	7.3 ± 3.5 (−1 to 16)	7.3 ± 2.8 (3–10)	0.016
Stiffness	1.7 ± 1.5 (0–5)	2.4 ± 1.5 (−1 to 5)	2.1 ± 1.2 (0–3)	0.095
Function	20.9 ± 12.4 (−1 to 48)	23.8 ± 12.2 (5–56)	22.7 ± 8.5 (12–39)	0.483

*HSS* Hospital for Special Surgery, *WOMAC* Western Ontario and McMaster Universities Arthritis Index

## Discussion

In the present study, the clinical improvement after HTO was not different regardless of the presence or absence of BME. The extent and location of BME could also affect the postoperative clinical results. Furthermore, patients with postoperative undercorrection showed less improvement in the WOMAC pain score compared with patients with acceptable corrections. Therefore, accurate alignment should be considered essential for achieving better clinical outcomes in patients with preoperative BME.

BME could be diagnosed in patients with trauma, stress fracture, osteonecrosis, and reactive change of OA [12, 14]. Especially BME on the medial compartment of the knee joint can be observed mostly in patients with varus deformities due to the effect of overloading on the subchondral bone. Felson et al. [18] reported that BME lesions were present in half of patients with pain, and pain was improved as the BME lesions disappeared. Furthermore, the risk of progression of knee OA could be increased by BME lesions [19]. However, in this study, the preoperative clinical scores were not different regardless of the presence or absence of BME. Moreover, the improvement of all clinical scores after HTO also was not different. Therefore, it was considered that BME was not significantly associated with preoperative clinical scores such as pain and postoperative clinical improvement after HTO.

Several results have reported the effect of BME lesions on the results after HTO. For instance, Kröner et al. [16] have reported BME reduction and clinical improvement. After HTO, postoperative valgus knee showed reduced edema, whereas neutral or varus knee showed either increased or unchanged BME in 40% of the patients and unsatisfactory clinical results. In this study, patients with undercorrection demonstrated a higher WOMAC pain score postoperatively compared with patients with acceptable corrections. HTO aims to correct mild valgus alignment postoperatively [6, 8, 20–22]. Postoperative varus correction may lead to recurrence of lower deformity, whereas overcorrection may cause lateral compartment knee OA [7, 23]. Therefore, accurate correction could be one of the most critical factors that affects postoperative results in patients with BME. Kim et al. [24] reported that the severity of BME is associated with the subsequent clinical outcomes. Especially, 1-year postoperative results are associated with the extent or intensity of BME, and clinical improvements were shown at 2 years postoperatively regardless of the severity of BME. In this study, the maximal diameter of BME could affect the postoperative clinical results, whereas the volume of BME was not associated with the postoperative clinical results. Yang et al. [25] reported on the midterm results after HTO in patients with tibial BME. The authors did not find any correlation between preoperative BME severity and postoperative clinical outcomes, and HTO could be considered an effective treatment option regardless of the degree of preoperative tibial BME. In this study, the position of BME, especially in patients with BME on both femur and tibia, was related to a lower Knee Society function score as opposed to patients with BME on either femur or tibia. Consequently, the position of BME could be associated with postoperative clinical outcomes.

In the present study, patients with BME did not exhibit poor postoperative clinical scores compared with patients without BME. More specifically, the clinical scores of patients without BME were not affected by the postoperative correction degrees. However, undercorrected BME patients showed lower WOMAC pain score improvements compared with well-corrected patients. Therefore, it can be suggested that accurate correction is an essential factor for achieving better clinical outcomes of HTO.

The limitations of this study include its small number of patients, retrospective design, and postoperative evaluation using clinical scores without using MRI to perform BME measurements. And, the status of cartilage, meniscus, or grade of OA were not considered. These variables could affect the postoperative clinical outcomes. Moreover, the mean postoperative mechanical axis was 58.3 ± 6.7%, which is lower than the target point (62.5%), and the high rate of patients with undercorrection after HTO could be the limitation of this study. A further long-term follow-up and prospective study with a large sample and evaluation of BME change should be performed.

## Conclusions

The clinical improvement after HTO in patients with varus and medial OA was not different regardless of the presence or absence of BME. However, accurate alignment should be considered essential for achieving better clinical outcomes in patients with preoperative BME.

## Data Availability

Not applicable.

## Abbreviations

HTO: High tibial osteotomy
BME: Bone marrow edema
WOMAC: Western Ontario and McMaster Universities Arthritis Index
OA: Osteoarthritis
MRI: Magnetic resonance imaging
IRB: Institutional review board
HSS: Hospital for Special Surgery
KS: Knee score
FS: Function score
